# A Simple and Sensitive RT-qPCR Technology for Rapid Detection of Porcine Reproductive and Respiratory Syndrome Virus

**DOI:** 10.3390/vetsci12010026

**Published:** 2025-01-07

**Authors:** Hongri Zhao, Xingyu Xiao, Yajuan Sun, Yang Chen, Yongzhe Zhang, Peng Li, Hui Jin, Ying Li, Rui Yin

**Affiliations:** 1 College of Veterinary Medicine, Jilin Agricultural University, Changchun 130118, China; zhaohongri@jlnku.edu.cn (H.Z.); caoqianyue@jlnku.edu.cn (Y.C.); zhangyongzhe@jlnku.edu.cn (Y.Z.); 2 College of Biological and Pharmaceutical Engineering, Jilin Agricultural Science and Technology University, Jilin 132101, China; xyxiao23@mails.jlu.edu.cn (X.X.); lp@jlnku.edu.cn (P.L.); jinhui0726@jlu.edu.cn (H.J.); 3 Department of Neurology, China-Japan Union Hospital of Jilin University, Changchun 130033, China; sunyaj@jlu.edu.cn; 4 Research and Development Center, Sairuisi Biotechnology (Jilin) Co., Ltd., Changchun 130102, China

**Keywords:** PRRSV, *M* gene, TaqMan RT-qPCR, fully pre-mixed reaction mixture, detection

## Abstract

To establish a rapid and sensitive detection method for the porcine reproductive and respiratory syndrome virus (PRRSV), gene-specific primers and a TaqMan probe were designed based on the *M* gene of PRRSV, and a new stable fully pre-mixed reverse transcription real-time fluorescence quantitative PCR (RT-qPCR) reaction mixture was developed. A simple and rapid RT-qPCR detection method for PRRSV was developed by optimizing nucleic acid amplification conditions. The results showed that the method was able to specifically detect PRRSV without cross-reactivity with the other 11 porcine susceptible viruses. The sensitivities of the assay were 3.12 × 10^0^ copies/μL and 10^0^ TCID_50_/μL for *M* gene and virus, respectively, and the repeatability and reproducibility (relative standard deviation, CV) of the assay were less than 2.5%. Based on the new fullly pre-mixed RT-qPCR reaction mixture, the RT-qPCR detection method may provide a new, simple, and rapid method for accurately detecting PRRSV.

## Simple Summary

Porcine reproductive and respiratory syndrome (porcine blue ear disease), a highly contagious disease caused by the porcine reproductive and respiratory syndrome virus (PRRSV), is capable of causing reproductive disorders in sows and respiratory syndrome in pigs of all ages, which severely affects the global pig industry. In this study, a generalized rapid test for PRRSV was established based on a new fully pre-mixed RT-qPCR reaction mixture that specifically detects PRRSV without cross-reactivity with other porcine susceptible viruses, with a minimum limit of 3.12 × 10^0^ copies/μL and 10^0^ TCID_50_/μL for the detection of PRRSV genes. For the precision assay, the relative standard deviation CV values were less than 2.5% for repeatability and reproducibility. The method was successful for rapid detection of 247 real samples. The fully pre-mixed RT-qPCR assay system established in this study not only simplified the operation steps but also shortened the assay time. This new assay meets the need for early and rapid detection of PRRSV and can provide an efficient, rapid, and sensitive detection method for PRRSV infection, thus laying the foundation for better prevention and control of the occurrence and spread of PRRS.

## 1. Introduction

Porcine reproductive and respiratory syndrome (PRRS), also known as swine blue ear disease [1,2], is an infectious disease caused by the porcine reproductive and respiratory syndrome virus (PRRSV) [3]. It is one of the most serious diseases threatening the swine industry [4]. PRRS is characterized by reproductive disorders in sows and respiratory symptoms in pigs of all ages as the main clinical signs [5,6,7]. Gestating sows mainly show symptoms such as abortion, stillbirth, and a decline in delivery rates, and pigs show severe respiratory symptoms. Breeding pigs have breathing difficulties and developmental delays, reduced libido, decreased semen quality, etc. The suppressed immune system of pigs infected with PRRSV makes them susceptible to other pathogens [8,9]. Infected pigs can transmit the virus through various routes, including feces, urine, saliva, and nasal secretions [10,11,12], contaminating feed [13,14,15], water, and the surrounding environment [16]. In addition, after sows are infected, the virus can be vertically transmitted through the placenta to the fetus, leading to arrested fetal growth, abortion, or the birth of a malformed piglet [11]. PRRS has caused significant economic losses to the global swine industry [2,4,17].

PRRSV is an enveloped, single-stranded, positive-sense RNA virus belonging to the genus Betaarterivirus and the family Arteriviridae [18]. It has a genome of approximately 15.4 kb and exhibits significant genetic and antigenic diversity [2,19,20]. Based on the viral genome sequence information, PRRSV is primarily divided into two types: PRRSV-1 (European strains, represented by the Lelystadvirus strain) and PRRSV-2 (American strains, represented by the VR-2332 strain) [21,22,23]. PRRSV-1 diversity is mainly distributed in Europe, including four genotypes (subtype I, subtype II, subtype III, subtype IV) [24]. In recent years, PRRSV-1 has been introduced into five non-European countries: the United States, Canada, South Korea, China, and Thailand. Currently, all PRRSV-1 strains isolated in China belong to subtype I [25]. PRRSV-1 can be further classified into four subgroups in China, including the BJEU06-1, Amervac, HKEU16, and NMEU09-1 strains [26]. Recent studies have shown that PRRSV-1 strains of the BJEU06-1 type are more prevalent in China than the other subgroups [27]. PRRSV-2 can be classified into nine genotypes (lineage 1–9). Lineage 1 contains strains such as NADc30 and JL580; lineage 2 contains strains such as 1205-GD-TTC and HB-1; lineage 3 contains low-pathogenicity strains such as QYYZ and GM2; lineage 4 contains vaccine-like strains such as JXA-R; lineage 5 contains strains such as VR-2332; lineage 6 contains isolates such as JSWA and SDEF1508; lineage 7 contains isolates such as GD-2011 and 15JX1; lineage 8 contains highly pathogenic strains such as TJ, JXA1-R, and TA-12 as well as classical strains such as CH-1a; and lineage 9 was found in Xinjiang in 2011 and subsequently found in Guangdong Province [24,25,28,29,30]. PRRSV-2 is now coexisting with multiple genotypes in China. Currently, there is no effective treatment for PRRS [31], which has a significant impact on the swine industry. Therefore, for the healthy development of the swine industry, early detection of PRRSV infections is crucial to prevent and block the spread of the disease.

Reverse transcription real-time fluorescence quantitative PCR (RT-qPCR) has the advantages of speed, specificity, and sensitivity [32,33] and is widely used in the early detection of PRRSV. Long et al. established a multiplex crystal digital RT-PCR detection method that can simultaneously detect the different strains of PRRSV-2 [34]. Qiu et al. established a fluorescence quantitative PCR method for detecting PRRSV-2 that can also differentiate classic-type, highly pathogenic, and NADc30-like PRRSV [6]. To meet the demand for PRRSV detection, some companies have developed PRRSV detection kits using fluorescence qPCR in China. Although qPCR technology has been widely applied to detect PRRSV, these PRRSV qPCR detection techniques are complicated to operate and time consuming, that is, these reported technologies need an operation step of mixing the qPCR master mix with primer and probe, which is cumbersome to operate; in addition, these qPCR technologies usually take 60–90 min to complete [6,35,36,37,38].

This study established a simple, rapid, and sensitive detection technology for PRRSV using RT-qPCR. By preparing a fully pre-mixed reaction mixture that incorporates all necessary components for amplification, the detection process is streamlined; the only requirement is the addition of the RNA template to the RT-qPCR system. The fully pre-mixed RT-qPCR detection system developed in this study not only simplifies the operational steps but also reduces the detection time, enabling completion within 30 min. This innovative detection method offers a valuable approach for the early and rapid identification of PRRSV in the swine industry.

## 2. Materials and Methods

### 2.1. Sample Source

This study collected 247 biological samples from six breeding pig farms in Jilin City, China. The samples included 207 random samples with unknown infection status (93 serum samples and 114 swab samples) as well as 30 samples that tested positive for PRRSV and 10 samples that were confirmed negative for PRRSV. The determination of positive and negative samples was conducted by the testing facilities of the pig farms. All collected samples were stored at −80 °C for future use.

### 2.2. Virus, Vaccines, and Recombinant Plasmids

PRRSV-1 strains (HKEU16 strain, HK3 strain, LV4.2.1 strain, R24 strain, and BEL/WVL/2018 strain) and PRRSV-2 strains (NADc30 strains, CH-1a strain, CH-1R strain, R98 strain, and JXA1-R strain) were obtained from Sairuisi Biotechnology (Jilin) Co., Ltd., Jilin, China, with a viral titer from 10^4.5^ TCID_50_/μL to 10^5.0^ TCID_50_/μL. The vaccines included classical swine fever vaccine (CSFV), porcine pseudorabies vaccine (PRV, HB-98 strain), swine transmissible gastroenteritis, porcine epidemic diarrhea and porcine rotavirus (G5 type) vaccine (TGEV& PEDV& PoRV), foot and mouth disease vaccine (FMDV, Type O, A dual—valent 3B protein epitope-deleted inactivated), swine influenza virus subtype H1N1 vaccine (SIV), porcine parvovirus vaccine (PPV), porcine circovirus type 2 vaccine (PCV2), and Japanese encephalitis vaccine (JEV). Detailed information on the vaccines is provided in Table A1.

The plasmid (100 ng/μL) containing the whole genomic sequence of the porcine circovirus type 3 29160 strain and a standard plasmid, PRRSV-*M*-Plasmid containing the *M* gene for the PRRSV NADc-30 strain (3.12 × 10^10^ copies/μL), were synthesized by General Biotechnology Co., Ltd. (Chuzhou, China).

### 2.3. Main Reagents and Instruments

The magnetic bead method virus DNA/RNA extraction kit was purchased from Sairuisi Biotechnology Co., Ltd. (Jilin, China). The HiScript II U^+^ One Step qRT-PCR Probe kit was purchased from Novozymes Biologics Co., Ltd. (Nanjing, China). The qRT-PCR kit [v6] (UDG System) was purchased from Cnpair Biotech Co., Ltd. (Hangzhou, China). The control reagent, a universal RT-qPCR detection kit for PRRSV (V20211001), was purchased from Beijing Anheal Laboratories Co., Ltd. (Beijing, China).

The materials used to verify the anti-interference capability of the PRRSV RT-qPCR detection method included endogenous interfering substances, such as blood, milk, tissue organs, and mucin, or exogenous interfering substances, such as feed residues, antibiotics, and antiviral drugs. The drug reagents used were amoxicillin for injection, tilmicosin premix, doxycycline hydrochloride-soluble powder, tylosin fumarate-soluble powder, ceftiofur sodium for injection, florfenicol powder, amantadine hydrochloride tablets, ribavirin granules, dexamethasone acetate tablets, and gentamicin sulfate. The detailed preparation method for the interference substances is provided in Table A2.

The fully automated nucleic acid extraction and purification instrument TGuide S32 was purchased from Tiangen Biochemical Technology Co., Ltd. (Beijing, China), the nucleic acid concentration determination instrument Nano Drop One was purchased from Thermo Fisher Scientific Co., Ltd. (Shanghai, China), and the real-time fluorescent quantitative PCR instrument Gentier 96R was purchased from Xi’an Tianlong Technology Co., Ltd. (Xian, China).; 7500 and StepOne Plus were purchased from Thermo Fisher Scientific Co., Ltd. (Shanghai, China).

### 2.4. Nucleic Acid Extraction

The viral RNA or DNA was extracted using the magnetic bead method viral RNA/DNA nucleic acid extraction kit, and then, the extracted RNA or DNA was stored at −80 °C.

### 2.5. Design and Screening of Specific Primer Probes for PRRSV

Based on the published full-genome sequences of the PRRSV-1 and PRRSV-2 representative strains (GenBank numbers: EU076704.1, KF287128.1, JF276433.1, KX668221.1, KC862570.1, KP860912.1, KY434184.1, MF346695.1, MK876228.1, MZ417465.1, OM893851.1, MN175678.1, JN654459.1, EF536003.1, JF802085.1, FJ548855.1, AF184212.1, EF536000.1, AF331831.1, EU807840.1, JN662424.1, MN648449.1, KP861625.1, MF326985.1, EF112445.1, AB288356.1, AF494042.1, and KF724404.1), two pairs of primers and probes were designed for the conserved region of *M* gene for PRRSV and then used for primer and probe selection. The primers and probes were synthesized by General Bioscience Co., Ltd. (Chuzhou, China). The primer and probe sequences are listed in Table 1.

### 2.6. Fully Pre-Mixed RT-qPCR Development of the System and Reaction Procedures

The PRRSV RT-qPCR detection system contains 5 μL of nucleic acid templates and 20 μL of the fully pre-mixed RT-qPCR detection system. The fully pre-mixed RT-qPCR system, including 12.5 μL PCR buffer, 1.5 enzyme mix (2.4 U/μL RNase inhibitor, 0.5 U/μL UNG enzyme, 16 U/μL high-efficient reverse transcriptase, and 0.15 U/μL rapid Taq DNA polymerase), 1.25 μL of primers and probe (0.4 μmol/L primer and 0.1 μmol/L probe), and ddH_2_O, was fixed to 20 μL.

To enhance the amplification efficiency of the reaction, the PCR enhancers 0.5 μg/mL bovine thrombin (BT), 1 ng/μL single-stranded binding protein (gp32), 2% dimethylsulfoxide (DMSO), 6 mmol/L dithiothreitol (DTT), and 0.5% sucrose were sequentially added to the fully pre-mixed system [39,40,41,42]. Following the identification of effective PCR enhancers, the concentration of each enhancer was optimized. Subsequently, the optimal concentration of each PCR enhancer was incorporated into the reaction system to evaluate potential synergistic effects. The template utilized for validation was PRRSV RNA (JXA1-R), and the primers and probes employed were those screened in Section 2.5.

The RT-qPCR reaction system contains 5 μL of nucleic acid template and 20 μL of RT-qPCR Master Mix. The RT-qPCR Master Mix includes 12.5 μL of PCR buffer (0.25 mol/L Tris pH 8.6, 50 mmol/L KCl, 24 mmol/L MgCl_2_, 0.25 mmol/L dNTPs and dUTPs, 1 μg/mL bovine thrombin, 8 mmol/L DTT, 2 ng/μL single-strand binding protein (gp32), 2.5% DMSO, and 0.5% sucrose), 4.5 μL of enzyme mix (2.4 U/μL RNAse inhibitor, 0.5 U/μL UNG enzyme, 16 U/μL high-efficient reverse transcriptase, and 0.15 U/μL rapid Taq DNA polymerase), 1.25 μL of primers and probe (0.4 μmol/L primer and 0.1 μmol/L probe), and 3.75 μL of RNase-Free H_2_O.

The RT-qPCR rapid reaction program was as follows: reverse transcription at 50 °C for 5 min, pre-denaturation at 95 °C for 5 min, followed by 45 cycles of denaturation at 95 °C for 5 s and annealing and extension at 60 °C for 15 s. Based on the amplification curve and cycle threshold (Ct) value, the judgment criteria were as follows: If the Ct value was ≤40, it was considered PRRSV positive. If the Ct value was between 40 and 45, the experiment was repeated. If the Ct value was still within this range or less than 40, the sample was considered PRRSV positive. If the Ct value was >45 or there was no amplification curve, it was considered negative for PRRSV.

### 2.7. Optimization of the PRRSV RT-qPCR System

To obtain the optimal RT-qPCR conditions, the concentrations of primers, probes, and annealing temperatures were optimized. Using standard plasmids, PRRSV-*M*-Plasmid (3.12 × 10^5^, 3.12 × 10^4^, and 3.12 × 10^3^ copies/μL) as the DNA templates, the primer concentrations were set at 0.2, 0.4, 0.6, and 0.8 μmol/L (the probe concentration was kept at 0.2 μmol/L) for optimization. After the optimal primer concentration was determined, the probe concentration was set at 0.1, 0.2, and 0.3 μmol/L for optimization. Finally, the annealing temperatures were set at 58 °C, 60 °C, and 62 °C for optimization. Each test was repeated thrice.

### 2.8. Comparison of the Fully Pre-Mixed RT-qPCR System with the Commercial Master Mix

In order to validate the effectiveness of the fully pre-mixed RT-qPCR (simply add the templates) method established in this study and the two conventional master mixes (both requiring stepwise addition of master mix, primer probe mixtures, and templates) for the detection of PRRSV, a comparative study of the amplification efficiency, R^2^, and assay reproducibility (CV) of the three methods was carried out, respectively. The reaction systems and reaction procedures for the two conventional master mixes were carried out according to the instructions.

### 2.9. Specific Assay

To verify the specificity of the test, the nucleic acids of PRRSV (JXA1-R strains) and other swine susceptible viruses (PRV, PPV, PCV2, PCV3, CSFV, JEV, TGEV, PEDV, PoRV, SIV, FMDV) were amplified using the newly developed assay. Double-distilled water was used as the negative control. The test was repeated thrice.

### 2.10. Sensitivity Assay

To determine the minimum detection limit of the test, a 10-fold serial dilution of PRRSV-*M*-Plasmid (3.12 × 10^8^ to 3.12 × 10^−1^ copies/µL) was used as the templates and detected using this RT-qPCR method. A standard curve was established to assess the amplification efficiency of RT-qPCR. This experiment was repeated thrice.

To further evaluate the minimum detection limit of the established RT-qPCR method for PRRSV, the PRRSV-2 JXA1-R strain (viral titers of 10^5.0^ TCID_50_/μL) was subjected to a 10-fold serial dilution up to 10^−1^ TCID_50_/μL, and the RNA samples were extracted. These RNA were detected using the newly developed RT-qPCR method. The tests were performed in three triplicates.

### 2.11. Inclusive Assay

To verify the universality of the primers and probes for the detection of different genotypes of the PRRSV strains, ten strains of PRRSV (HKEU16 strain, HK3 strain, LV4.2.1 strain, R24 strain and BEL/WVL/2018 strain, CH-1a strain, CH-1R strain, R98 strain, NADc30 strains, and JXA1-R strain) were chosen, and each of these strains was diluted to the lowest limit of detection of the test and then detected using the newly developed assay, and finally, the inclusiveness of the test was assessed.

### 2.12. Precision Assay

The repeatability of the tests was assessed. Three different concentrations (3.12 × 10^5^, 3.12 × 10^4^, and 3.12 × 10^3^ copies/μL) of PRRSV-M-Plasmid were used as templates for RT-qPCR amplification. In the intra-assay, four replicates of each template concentration were amplified using the same batch of RT-qPCR reaction solutions. Five batches of RT-qPCR reaction solutions were prepared five working days per week, and three template concentrations were detected. The coefficient of variation (CV) was calculated as follows: CV=Standard deviation÷mean×100%.

In addition, to assess the reproducibility of the test, three different titers (10^3.0^ TCID_50_/μL, 10^2.0^ TCID_50_/μL, 10^1.0^ TCID_50_/μL) of PRRSV JXA1-R were used as templates for RT-qPCR amplification. And this test was performed at three laboratories [Molecular Diagnostics Science and Technology Innovation Center of Jilin Agricultural Science and Technology University, Molecular Testing Laboratory of Jilin Agricultural University, and Research Laboratory of Sairuisi Biotechnology (Jilin) Co., Ltd.]. And three brands of qPCR instruments (Xi’an Gentier 96R Tianlong, Xi’an, China, ABI 7500 and ABI StepOne Plus, USA) with three batches of reagents were performed by three different operators. Finally, the standard deviation (SD) and relative standard deviation (CV) of the Ct values were calculated to assess the reproducibility of the test.

### 2.13. Anti-Interference Test

An experimental scheme for interference was designed based on the type of clinical sample and the potential interfering factors in the samples. Endogenous interfering substances were used, including blood, tissue organs (such as the liver), and mucin, whereas exogenous interfering substances included antibiotics, antiviral drugs, and microecological preparations. A total of 100 μL of JXA1-R virus (viral titers of 10^1.0^ TCID_50_/μL) was mixed with 100 μL of endogenous or exogenous interfering substances, with ddH_2_O used for the negative control. And then, the RNA samples were extracted. Then, 5 μL of nucleic acids from the mixture was used for RT-qPCR amplification.

### 2.14. Detection of the Real Samples

To evaluate the detection capacity of the newly developed method in real samples, a total of 247 samples (Section 2.1) were analyzed in this test. To verify the accuracy of the test further, 247 samples were tested using the control reagent.

## 3. Results

### 3.1. Screening of Prime Probes Specific for PRRSV

**Figure 1:**
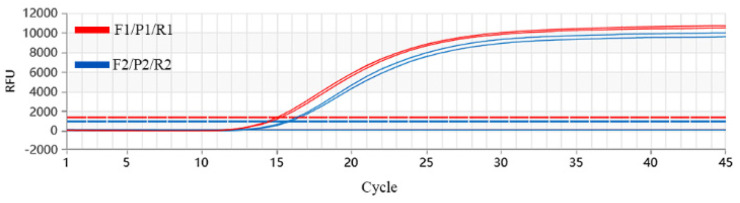
Screening of primers and probes for PRRSV. The red lines are the amplification results of F1R1/P1/. The blue lines are the amplification results of the F2/R2/P2/.

Using JXA1-R strain RNA as the templates, two sets of primers and probes were selected using the fully pre-mixed RT-qPCR mixture. The results showed that typical s-type amplification curves were obtained for both sets of primers and probes. Compared with the second set of primers and probes, the first set showed smaller Ct values and a higher plateau phase on the amplification curves (Figure 1). Therefore, the first set of primers and probe were used in subsequent experiments.

### 3.2. Effect of PCR Enhancers for Fully Pre-Mixed RT-qPCR Systems

Supplementary Figure S1 illustrates the optimal concentrations of BT, gp32, DMSO, DTT, and sucrose that enhance PCR performance in the reaction system, which were found to be 1 μg/mL, 2 ng/μL, 2.5%, 8 mmol/L, and 0.5%, respectively. Building on this observation, we simultaneously introduced all five PCR enhancers into the fully pre-mixed reaction system, leading to a further reduction in the Ct value of PRRSV (Table 2). This finding indicates a potential synergistic effect among the PCR enhancers.

### 3.3. Optimization of the PRRSV RT-qPCR Reaction System

**Figure 2:**
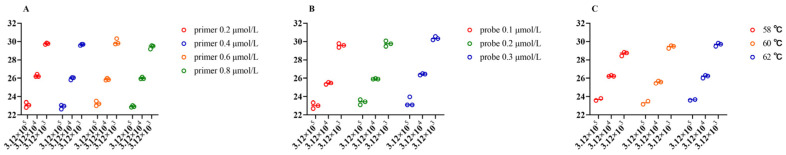
Optimization of PRRSV RT-qPCR reaction system. (**A**) Optimization results of primer concentration. The red markings are the results with the primer of 0.2 μmol/L. The blue markings are the results with the primer of 0.4 μmol/L. The yellow markings are the results with the primer of 0.6 μmol/L. The green markings are the results with the primer of 0.8 μmol/L. (**B**) Optimization results of probe concentration. The red markings are the results with the probe of 0.1 μmol/L. The green markings are the results with the probe of 0.2 μmol/L. The blue markings are the results with the probe of 0.3 μmol/L. (**C**) Optimization results of denaturation temperature. The red markings are the results with the denaturation temperature of 58 °C. The yellow markings are the results with the denaturation temperature of 60 °C. The blue markings are the results with the denaturation temperature of 62 °C.

Optimization results for the PRRSV RT-qPCR reaction system indicate that fluorescence detection signal intensity was low when the primer concentration was 0.2 μmol/L, and there was no significant difference in detection Ct values and amplification efficiency when the primer concentrations were 0.4, 0.6, and 0.8 μmol/L. Therefore, 0.4 μmol/L of the primer was used for subsequent studies. The amplification curves showed a good linear relationship when the probe concentration was set at 0.1 and 0.3 μmol/L; however, the amplification efficiency was higher when the probe concentration was 0.1 μmol/L. Thus, the final probe concentration was determined as 0.1 μmol/L. When the denaturation temperature was 58 °C, the amplification reaction showed a good linear relationship, and the amplification efficiency was within the acceptable range. Therefore, 58 °C was determined to be the optimal annealing temperature (Figure 2 and Table 3).

The final detection reaction system was set as follows: primer concentration 0.4 μmol/L, probe concentration 0.1 μmol/L, and optimal annealing temperature 58 °C.

### 3.4. Comparison of the Amplification Effect of Fully Pre-Mixed RT-qPCR System with Commercial Master Mix

The 3.12 × 10^5^, 3.12 × 10^4^, and 3.12 × 10^3^ copies/μL of the PRRSV-M-Plasmid were tested using an established fully pre-mixed RT-qPCR system and two commercial master mixes. The results are shown in Table 4. The amplification efficiency, R^2^, and test repeatability of the fully pre-mixed RT-qPCR system are 101.840%, 0.999, and CV < 1, respectively, all of which outperform the two commercial master mixes.

### 3.5. PRRSV RT-qPCR Specificity Test

**Figure 3:**
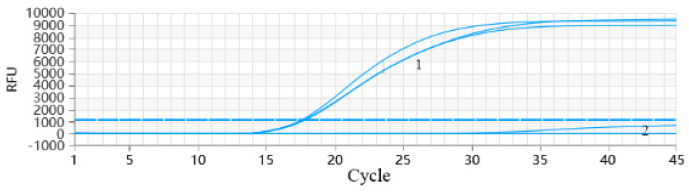
Specificity of PRRSV RT-qPCR. 1: detection result of PRRSV (JXA1-R strains). 2: detection result of PRV, PPV, PCV2, PCV3, CSFV, JEV, TGEV, PEDV, PoRV, SIV, FMDV, and negative control.

To assess the specificity of the test, the nucleic acids of PRRSV and other common pig susceptible viruses (PRV, PPV, PCV2, PCV3, CSFV, JEV, TGEV, PEDV, PoRV, SIV, FMDV) were amplified using RT-qPCR. The results showed that only the PRRSV RNA was positive. The other nucleic acid samples tested negative. Therefore, the PRRSV RT-qPCR detection method had good detection specificity (Figure 3).

### 3.6. The PRRSV RT-qPCR Sensitivity Test and the Establishment of the Standard Curve

**Figure 4:**
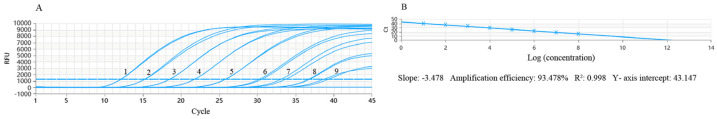
Sensitivity and standard curve of PRRSV RT-qPCR. (**A**) Sensitivity of PRRSV RT-qPCR. The curves 1–9: PRRSV-M-Plasmid concentrations from 10^8^ copies/μL to 10^0^ copies/μL. (**B**) Standard curve of PRRSV RT-qPCR. The PRRSV-M-Plasmid concentrations from 10^8^ copies/μL to 10^1^ copies/μL, the slope is −3.478, amplification efficiency is 93.478%, R^2^ is 0.998, and Y-axis intercept is 43.147.

To determine the sensitivity of the assay, we found that the assay could successfully detect 3.12 × 10^8^–3.12 × 10^0^ copies/μL of the PRRSV-*M*-Plasmid. The average Ct value was 38.99 when the gene copy number was 3.12 × 10^0^ copes/μL, and the assay failed to detect the target gene when the gene copy number was 3.12 × 10^−1^ copies/μL (Figure 4). Therefore, the minimum detection limit of the assay for the target gene was 3.12 × 10^0^ copies/μL. The assay showed good linearity on the DNA template (range from 3.12 × 10^8^ to 3.12 × 10^1^ copies/μL), with an amplification efficiency of 93.478%, and the correlation coefficient (R^2^) was 0.998.

**Figure 5:**
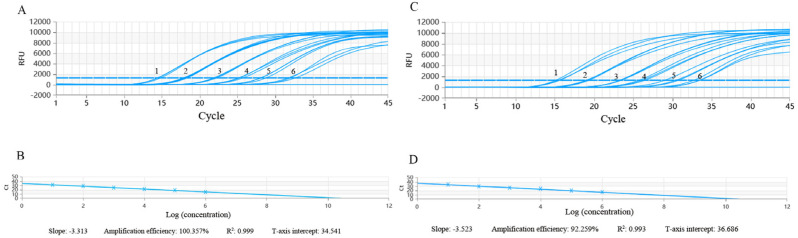
Detection limit testing. (**A**): The detection limit of RT-qPCR for PRRSV. The curves 1–6: virus titers from 10^5.0^ TCID_50_/μL to 10^1.0^ TCID_50_/μL. (**B**): Standard curve of RT-PCR for PRRSV. Virus titers from 10^5.0^ TCID_50_/μL to 10^1.0^ TCID_50_/μL, the slope is −3.313, amplification efficiency is 100.357%, R^2^ is 0.999, and Y-axis intercept is 34.541. (**C**): The detection limit of commercial qPCR kit for PRRSV. The curves 1–6: Virus titers from 10^5.0^ TCID_50_/μL to 10^1.0^ TCID_50_/μL. (**D**): Standard curve of commercial qPCR kit for PRRSV. Virus titers from 10^5.0^ TCID_50_/μL to 10^1.0^ TCID_50_/μL, the slope is −3.523, amplification efficiency is 92.259%, R^2^ is 0.993, and Y-axis intercept is 36.686.

Furthermore, the detection limit of RT-PCR for the PRRSV was 10^0^ TCID_50_/μL_._ The average Ct value was 32.01 when the viral load was 10^0^ TCID_50_/μL. The assay showed a good linearity on these RNA templates (10^5.0^ TCID_50_/μL–10^1.0^ TCID_50_/μL), with an amplification efficiency of 100.357%, and the correlation coefficient (R^2^) was 0.999. In addition, the detection limit of the commercial qPCR kits for PRRSV was also up to 10^0^ TCID_50_/μL (Figure 5).

### 3.7. PRRSV RT-qPCR Inclusive Assay

**Figure 6:**
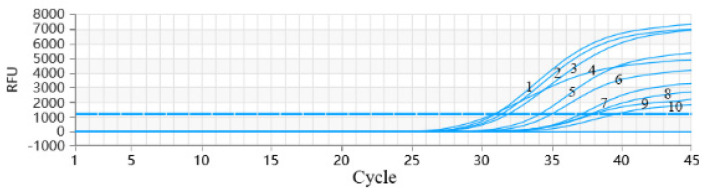
Inclusive of PRRSV RT-qPCR. The curves 1–10: NADc30 strains, JXA1-R strain, CH-1a strain, R98 strain, CH-1R strain, HKEU16 strain, LV4.2.1 strain, HK3 strain, R24 strain, and BEL/WVL/2018 strain.

Using this newly developed test, the capability of detecting different genotypes of PRRSV strains (Section 2.10) was studied. The results showed that all 10 genotypes of the PRRSV strains were successfully detected (Figure 6), indicating that the specific primers and probes can be applied for the detection of the 10 genotypes of the PRRSV strain.

### 3.8. PRRSV RT-qPCR Precision Assay

Three concentrations (3.12 × 10^5^, 3.12 × 10^4^, and 3.12 × 10^3^ copies/μL) of PRRSV-*M*-Plasmid were used for DNA templates and then amplified with the RT-qPCR assay. The results showed that the CV for the intra-batch and inter-batch repeatability tests was less than 0.5% (Table 5), indicating that the newly developed RT-qPCR assay had good repeatability.

Meanwhile, in order to assess the reproducibility of the test, the reproducibility test was performed in three laboratories with different equipment, different operators, and different batches of reagents. The results showed that the SD value of the assay was less than 1.0, and the CV value was less than 2.5%, indicating that the method has good reproducibility (Table 6).

### 3.9. PRRSV RT-qPCR Anti-Interference Test

Simulated clinical samples were prepared by mixing the JXA1-R viral strain (viral titers of 10^1.0^ TCID_50_/μL) with endogenous or exogenous interfering substances. Next, the nucleic acids were extracted and amplified using the newly developed RT-qPCR assay to assess the impact of potential interfering substances on RT-qPCR amplification. The results showed that exogenous interfering substances, such as antibiotics, antiviral drugs, probiotics, and feed, had no effect on the RT-qPCR detection, and endogenous interfering substances in tissue samples (such as the liver and lungs) and fecal samples had no significant impact on the assay. However, milk, blood, and mucin inhibited the RT-qPCR reaction, and the average Ct value increased 2–5 compared to the control group (Table 7). This may be due to the fact there are several PCR inhibitors that are not completely removed from these samples during nucleic acid extraction that may inhibit the activity of the RT-qPCR reaction.

### 3.10. Detection of Real Samples

A total of 247 real samples were tested using the newly developed method. The results showed that PRRSV was detected as positive in the thirty PRRSV-infected samples. In addition, there were 14 samples that were also detected as positive in 207 of the unknown status samples, with a positive detection rate of 6.8% (5.3% in serum samples, 1.5% in swabs), and the detection was negative for the 10 known PRRSV-negative samples.

To verify the accuracy of the test, these samples were also subject to retest using the control reagent. PRRSV was also detected as positive in the thirty PRRSV-infected samples. In addition, there were 11 samples that were detected as positive in 207 of the unknown status samples, with a positive detection rate of 5.3% (5.3% in serum samples, 0.0% in swabs) (Table 8), and the detection was negative for the 10 known PRRSV-negative samples. For the three samples that tested positive by the method established in this study and negative by the control reagent, the PCR products were purified and further confirmed by Sanger gene sequencing [sequencing was performed by Shenggong Bioengineering Co. Ltd. (Shanghai, China)]; the gene sequencing results showed that these samples were indeed positive for PRRSV (Supplementary Figure S2). This result is further proof that the newly developed assay has a higher detection rate for real clinical samples, and it can be used for the rapid detection of PRRSV.

## 4. Discussion

PRRSV poses a significant economic challenge to the global swine industry [43]. In both commercial swine farms and small-scale breeding operations, PRRSV is increasingly demonstrating a pattern of co-existence among multiple strains; this phenomenon not only leads to recombination among different strains but also causes the emergence of new virus strains [43,44,45,46]. Consequently, developing reliable detection methods is crucial for effective prevention and control of PRRS.

The RT-qPCR technology is widely used for the early diagnosis of animal diseases. Currently, qPCR assays have been developed for different target genes of PRRSV, such as Nsp2 genes, GP5 genes, and N genes [7,34,47,48,49], and these methods are able to detect PRRSV in virus samples such as blood, tissue, and environmental samples [50]. In existing RT-qPCR assay techniques, primers, probes, and enzyme mixtures are typically stored in separate tubes and mixed sequentially prior to use. This practice is necessary because the premature mixing of multiple components can lead to instability within the reaction system, which may subsequently reduce the sensitivity of the assay and compromise the accuracy of the results. This study is different from previously reported methods. In this study, the primers and probes were designed based on *M* gene of PRRSV, and a low-cost, easy-to-operate, fully pre-mixed reaction RT-qPCR system for PRRSV was established. We also compared the fully pre-mixed reaction system with a previously reported PRRSV RT-qPCR detection system. This comparison focused on several key parameters, including assay time, sample addition steps, target gene selection, sensitivity, and specificity. As detailed in Table A3, our study only uses one step of adding RNA templates into the fully pre-mixed reaction mixture (stored as enzyme, primers, and probe in one solution), and the RT-qPCR can be completed in 30 min. Additionally, in this study, the sensitivity for the target gene of the assay was up to 3.12 × 10^0^ copies/μL; the sensitivity of this test was ten times higher than the other technologies reported previously [47,51,52]. Furthermore, the reagent cost for establishing the detection method in this study is 2 dollars, and the cost of similar technology development products on the market is also between 2 and 3 dollars.

PCR enhancers play a crucial role in the polymerase chain reaction (PCR), primarily by significantly improving the efficiency and specificity of the reaction. These enhancers operate by enhancing enzyme stability, facilitating primer–template binding, and reducing non-specific amplification. In this work, it was investigated whether the addition of BT, gp32, DMSO, DTT, and sucrose into the reaction system would augment the efficiency and specificity of PCR. The results indicated that all five PCR enhancers positively influenced the reaction system. During the optimization of enhancer concentrations, it was observed that using each enhancer at concentrations exceeding a reasonable range could inhibit the PCR reaction (Figure 2). Additionally, the potential synergistic effects among the the enhancers were investigated, and we found evidence of synergy, which effectively improved PCR amplification efficiency. However, the mechanisms by which PCR enhancers act synergistically require further investigation. Consequently, the judicious use of PCR enhancers and careful consideration of their concentrations are critical when employing PCR for gene amplification.

In this test, the specificity, sensitivity, inclusiveness, and anti-interference test as well as the ability to detect clinical samples of the assay were investigated. The results showed that the method can specifically detect PRRSV, and no cross-reaction with the other eleven viruses was observed. The sensitivity of the standard plasmid was 3.12 × 10^0^ copies/μL, which is 10 times higher than similar techniques [47,51,52], and the lowest detection limit for viral load was 10^0^ TCID_50_/μL, which is more sensitive compared with the methods established previously [53,54,55]. This test also has good repeatability and reproducibility; the SD value was less than 1.0, and the CV value was less than 2.5%. The results of the interference resistance test demonstrated that the RT-qPCR amplification effect of milk, blood, and mucin samples was relatively poor. Future research will concentrate on developing pretreatment methods for samples rich in blood, milk, and mucin to improve the stability and reliability of the detection method we have already established. Potential sample pretreatment strategies might involve optimizing the existing methods, using specific reagents, or applying filtration techniques to purify nucleic acids. This RT-qPCR method was able to detect PRRSV in real samples and provides a higher accurate positive detection rate (6.8%) than control reagents (5.3%) in unknown infection status samples. In comparison to the digital crystal detection method established by Yang et al. and Shi et al. [37,56], there is a need for improved detection sensitivity. While the current results of digital crystal detection are promising, it is worth noting that digital droplet PCR (dPCR) is more expensive than qRT-PCR detection and has not yet been implemented for large-scale screening of clinical samples.

Although the newly developed PRRSV RT-qPCR demonstrates technological advancements compared to previous detection systems [57,58], the newly developed assay still depends on traditional multi-step nucleic acid extraction prior to PCR amplification, which constrains its effectiveness for pathogen detection in the field. Future research will focus on investigating direct amplification qPCR as a potential solution for field-based PRRSV detection.

## 5. Conclusions

This study established an RT-qPCR detection method for PRRSV. This test has specificity, sensitivity, and repeatability, and as a new detection method for PRRSV, it may provide a new approach for the early and rapid detection of PRRSV in the swine industry.

## Figures and Tables

**Table 1:** Primer and probe sequences.

Primer and Probe	Sequence of 5′-3′	Fragment Length (bp)
PRRSV-F1	5′-TACATTCTGGCCCCTGCCCA-3′	196 bp
PRRSV-R1	5′-CCTCACCACTTGGAACAATTTATAC-3′	
PRRSV-P1	6-FAM-TGCCACCCAACACGAGGC-MGB	
PRRSV-F2	5′-GGAATGGCCAGCCAGTCAA-3′	122 bp
PRRSV-R2	5′-TTCTTTTTAGGCCTCTTCGGGGTAA-3′	
PRRSV-P2	6-FAM-AGCTGTGCCAAATGCTG-MGB	

**Table 2:** Determination of enhancers in fully pre-mixed RT-qPCR system.

Enhancer	Ct Value			Ct Mean	SD
Control	17.160	16.762	17.379	17.100	0.255
BT	16.293	15.902	16.027	16.074	0.163
gp32	14.102	13.746	13.879	13.909	0.146
DMSO	14.176	14.121	14.436	14.244	0.137
DTT	16.395	16.277	16.057	16.243	0.146
Sucrose	16.285	16.739	16.012	16.345	0.299
Total	14.488	13.908	13.871	14.089	0.244

Note: BT: bovine thrombin, gp32: single-stranded binding protein, DMSO: dimethylsulfoxide, DTT: dithiothreitol. Total: All five enhancers were added. Control: The enhancers were not added.

**Table 3:** Optimization of PRRSV RT-qPCR reaction system.

Project	Parameter	Ct Average Value (3.12 × 10^5^/3.12 × 10^4^/ 3.12 × 10^3^ copies/μL)	Amplification Efficiency/R^2^
Primer concentration (μmol/L)	0.2	22.66/25.92/29.45	97.006%/R^2^ = 1.000
	0.4	22.96/25.99/29.76	99.580%/R^2^ = 0.997
	0.6	23.40/26.04/30.13	98.303%/R^2^ = 0.985
	0.8	23.18/26.27/29.68	102.977%/R^2^ = 0.999
Probe concentration (μmol/L)	0.1	22.60/25.29/29.09	102.862%/R^2^ = 0.992
	0.2	22.53/24.89/28.96	104.790%/R^2^ = 0.977
	0.3	22.75/25.73/29.61	98.619%/R^2^ = 0.993
Annealing temperature (°C)	58	23.69/26.05/28.61	97.676%/R^2^ = 0.998
	60	23.33/25.58/29.43	112.743%/R^2^ = 0.978
	62	23.64/26.19/29.68	114.306%/R^2^ = 0.991

**Table 4:** Comparison of the detection effects of fully pre-mixed RT-qPCR and commercial master mix.

RT-qPCR System	Amplification Efficiency	R^2^	Repeatability					
			3.12 × 10^5^		3.12 × 10^4^		3.12 × 10^3^	
			Ct Mean	CV (%)	Ct Mean	CV (%)	Ct Mean	CV (%)
A	101.840%	0.999	21.715	0.407%	25.344	0.458%	28.813	0.671%
B	83.677%	0.960	24.203	4.086%	29.324	3.240%	31.770	0.243%
C	90.050%	0.997	23.789	1.436%	27.434	6.242%	30.574	5.276%

Note: A: Fully pre-mixed RT-qPCR assay system established in this study. B: HiScript II U^+^ One Step qRT-PCR Probe kit [Novozymes Biologics (Nanjing) Co., Ltd., Nanjing, China]. (Non-fully pre-mixed reagent). C: qRT-PCR kit [v6] (UDG System) [Cnpair Biotech (Hangzhou) Co., Ltd., Hangzhou, China]. (Non-fully pre-mixed reagent).

**Table 5:** Repeatability of PRRSV RT-qPCR assay.

Concentration (copies/µL)	Intra-Batch Assay		Inter-Batch Assay	
	Ct Mean ± SD	CV (%)	Ct Mean ± SD	CV (%)
3.12 × 10^5^	21.43 ± 0.08	0.150%	21.54 ± 0.08	0.369%
3.12 × 10^4^	25.32 ± 0.07	0.268%	25.38 ± 0.06	0.248%
3.12 × 10^3^	28.86 ± 0.02	0.081%	28.84 ± 0.06	0.201%

**Table 6:** Reproducibility of PRRSV RT-qPCR assay.

Dates	Viral Titers (TCID_50_/μL)	Xi’an Gentier 96R	ABl 7500	ABl StepOne Plus	SD	CV %
		Ct Value				
6.24	10^3.0^	21.99/21.85/21.90	21.48/21.45/21.39	21.02/21.11/21.29	0.35	1.61%
6.25		21.76/21.62/21.66	22.08/21.82/21.96	21.11/21.02/21.30	0.37	1.72%
6.27		21.61/21.62/21.80	21.79/21.72/21.90	21.20/21.22/21.10	0.30	1.40%
6.28		21.40/21.42/21.49	21.72/21.82/21.64	21.21/21.02/21.24	0.26	1.21%
6.29		21.61/21.58/21.61	21.66/21.55/21.66	20.98/21.01/21.10	0.29	1.37%
6.24	10^2.0^	25.81/25.62/25.44	24.80/24.69/24.87	24.67/24.44/24.64	0.49	1.95%
6.25		25.09/25.10/25.20	25.97/25.68/25.85	24.54/24.42/24.35	0.61	2.47%
6.27		24.76/24.89/24.78	25.17/25.23/25.23	24.70/24.90/24.64	0.23	0.94%
6.28		24.74/24.71/24.86	25.35/25.36/25.10	25.02/25.10/24.87	0.24	0.97%
6.29		24.91/25.01/24.88	25.23/25.50/25.31	26.00/25.80/25.81	0.42	1.65%
6.24	10^1.0^	28.81/28.78/28.71	28.68/28.67/28.42	28.22/28.22/28.22	0.25	0.89%
6.25		28.49/28.46/28.61	28.46/28.50/28.40	28.13/28.14/28.23	0.17	0.60%
6.27		28.37/28.41/28.30	28.41/28.43/28.50	28.09/28.11/28.09	0.16	0.57%
6.28		28.20/28.17/28.20	28.72/28.60/28.66	28.26/28.52/28.25	0.22	0.79%
6.29		28.28/28.21/28.35	29.48/29.48/29.40	29.28/29.31/29.31	0.55	1.92%

**Table 7:** Interference analysis of PRRSV RT-qPCR assay.

	Experimental Group													Control Group	
	Endogenous Interfering Substances (1–5)					Exogenous Interfering Substances (6–13)								Positive	Negative
	1	2	3	4	5	6	7	8	9	10	11	12	13	14	15
T1	28.09	28.35	28.42	28.41	29.06	28.36	29.50	29.01	28.88	29.21	32.40	32.05	30.93	28.48	--
	28.87	28.77	28.39	28.50	28.63	28.57	28.86	29.50	28.52	29.36	32.84	32.62	31.01	28.25	--
T2	29.04	28.58	27.83	28.55	29.01	28.48	29.83	28.62	29.47	29.80	33.01	31.76	31.30	27.95	--
	28.46	28.94	21.43	29.77	29.31	28.48	29.39	29.37	28.70	29.50	32.55	32.29	30.52	28.67	--

Note: T1 and T2 represent the repeat detection for the same sample. Experimental groups 1–5 are exogenous interfering substances, and experimental groups 6–13 are endogenous interfering substances. Group 1: Ceftiofur sodium for injection + Fluphenicol powder + Telmicocin pre-mix + normal saline; Group 2: Amoxicillin for injection + Dxycycline hydrochloride-soluble powder + Tamrocin-soluble powder + normal saline; Group 3: Dexamethasone acetate tablets + Gentamicin sulphate + normal saline; Group 4: Ribavirin granules + Amantadine hydrochloride tablets + normal saline; Group 5: Feed residue treatment solution; Group 6: Throat swab treatment solution; Group 7: Liver tissue treatment solution; Group 8: Lung tissue treatment solution; Group 9: Intestinal tissue treatment solution; Group 10: Stool swab treatment solution; Group 11: Milk treatment solution; Group 12: whole blood; Group 13: mucin + normal saline. Group 14 is the positive control, and Group 15 is the negative control. Of these, the preparation of the interfering substances is shown in Table A2.

**Table 8:** Performance of PRRSV RT-qPCR and control kit for clinical samples.

Clinical Samples (247)	PRRSV RT-qPCR	Control Kits
Samples (known PRRSV positivity 30)	30/30	30/30
Samples (known PRRSV negative 10)	0/10	0/10
Blood serum samples (93)	11/93	11/93
Swab samples (114)	3/114	0/114
Positive detection rate	14/207 (6.8%)	11/207 (5.3%)

Note: Porcine Reproductive and Respiratory Syndrome Virus Universal Real-Time Fluorescence RT-PCR Kit was purchased from Beijing Anheal Laboratories Co., Ltd. The reaction system and reaction procedures were carried out according to the instructions of the kit.

## Data Availability

The data that support the findings of this study are available from the corresponding author upon reasonable request.

## Supplementary Materials

The following supporting information can be downloaded at: https://www.mdpi.com/article/10.3390/vetsci12010026/s1, Figure S1: Optimization results of enhancer concentration in full pre-mix system; Figure S2: Results of the Sanger gene sequencing of the samples PCR products; Figure S3: Optimization of the PRRSV RT-qPCR reaction system. Figure S4: Sequence alignment results of PRRSV.

## Appendix A

**Table A1:** Vaccine details.

Name	Specifications	Production Batch Number	Source
Swine Epidemic Encephalitis Vaccine, live (Strain SA14-14-2)	10 copies/bottle	20220721	Wuhan Keda Biotechnology Co., Ltd. (Wuhan, China)
Porcine Parvovirus vaccine, inactivated (Strain WH-1)	20 mL/bottle	20220817	Wuhan Keda Biotechnology Co., Ltd. (Wuhan, China)
Swine Influenza Virus Subtype H1N1 Vaccine, inactivated (Strain TJ)	20 mL/bottle	20220502	Wuhan Keda Biotechnology Co., Ltd. (Wuhan, China)
Swine Pseudorabies Vaccine, live (Strain HB-98)	10 copies/bottle	2212022	Zhongmu Industry Co., Ltd. (Shanghai, China)
Classical Swine Fever Live Vaccine (Cell-Derived) (CVCC AV1412 Strain)	10 copies/bottle	2201011	Zhongmu Industry Co., Ltd. (Shanghai, China)
Porcine circovirus disease type 2 vaccine, inactivated (Strain SH)	20 mL/bottle	072110006	PLeiro Biotechnology Engineering Co., Ltd. (Wuhan, China)
Swine Transmissible Gastroenteritis, Porcine Epidemic Diarrhea and Porcine Rotavirus (GP5 type) Vaccine, live (Strain huadu + Strain CV777 + Strain NX)	1 copies/bottle	2022020	Harbin Weike Biotechnology Co., Ltd. (Harbin, China)
Foot-and-mouth disease type O and A bivalent 3B protein epitope deletion vaccine, inactivated (O/rV-1 Strain + A/rV-2 Strain)	20mL/bottle	0AA220618	Inner Mongolia Biwei Antai Biotechnology Co., Ltd. (Hohhot, China)
Haemophilus parasuis Disease Quadrivalent Propolis Vaccine, Inactivated (Type 4 SD02 Strain + Type 5 HN02 Strain + Type 12 GZ01 Strain + Type13 JX03 Strain)	20 mL/bottle	202209003	Shandong Huahong Biotechnology Engineering Co., Ltd. (Binzhou, China)
Swine Staphylococcus Bee Propolis Inactivated Vaccine (Swine Staphylococcus serogroup C BHZZ-L1 Strain + Swine Staphylococcus type 2 BHZZ-L4 Strain).	20 mL/bottle	202209009	Shandong Huahong Biotechnology Engineering Co., Ltd. (Binzhou, China)
Piglet Enterotoxigenic *E. coli* Disease Tri-valent Inactivated Vaccine (containing K88, K99, 987P flagellar antigens)	10 mL/bottle	202209006	Shandong Huahong Biotechnology Engineering Co., Ltd. (Binzhou, China)
Porcine Mycoplasmal Pneumonia Inactivated Vaccine (Strain J)	20 mL/bottle	20220808	Shandong Huahong Biotechnology Engineering Co., Ltd. (Binzhou, China)
Triple Live Vaccine for Classical Swine Fever, Swine Erysipelas and Pasteurellosis in Pigs (Cell-Derived + G4T10 Strain + EO630 Strain)	10 copies/bottle	2208037	Zhongmu Industry Co., Ltd. (Shanghai, China)
Porcine Atrophic Rhinitis Inactivated Vaccine (Bordetella pertussis JB5 Strain)	20 mL/bottle	20220602	Wuhan Keda Biotechnology Co., Ltd. (Wuhan, China)
Triple-valent Inactivated Vaccine for Porcine Contagious Pleuropneumonia (Serotype 1 9901 Strain, Serotype 2 XT9904 Strain, Serotype 7 GZ9903 Strain)	20 mL/bottle	20220706	Wuhan Keda Biotechnology Co., Ltd. (Wuhan, China)

**Table A2:** Preparation of mock samples for anti-interference test.

	Experimental Group				
Exogenous Dry Perturbation Material	Constituent Content	Actual Dosage (μL)	Endogenous Dry Perturbation Material	Constituent Content	Actual Dosage (μL)
Group 1	Ceftiofur sodium for injection (300 μL)	100	Group 6	Throat swab treatment solution *	100
	Fluphenicol powder (30 μL)				
	Telmicocin pre-mix (30 μL)		Group 7	Liver tissue treatment solution *	100
	normal saline (240 μL)				
Group 2	Amoxicillin for injection (30 μL)	100	Group 8	Lung tissue treatment solution *	100
	Dxycycline hydrochloride-soluble powder (150 μL)				
	Tamrocin-soluble powder (150 μL) normal saline (300 μL)		Group 9	Intestinal tissue treatment solution *	100
Group 3	Dexamethasone acetate tablets (2.5 μL)	100	Group 10	Stool swab treatment solution *	100
	gentamicin sulphate (30 μL)				
	normal saline (567.5 μL)		Group 11	Milk treatment solution *	100
Group 4	Ribavirin granules (10 μL)	100			
	Amantadine hydrochloride tablets (15 μL)		Group 12	whole blood *	100
	normal saline (575 μL)				
Group 5	Feed residue treatment solution *	100	Group 13	mucin (270 μL) normal saline (270 μL)	100
	**control group**				
**positive control**	**constituent content**		**negative control**	**constituent content**	
Group 14	Virus titers 10^1.0^ TCID_50_/μL (100 μL) ddH_2_O (100 μL)		Group 15	ddH_2_O (200 μL)	

* Note on sample preparation methods: Feed: Take 5 g of feed sample and add 5 mL of PBS to soak for 10 min, periodically kneading and grinding during this time, at 12,000 rpm/min. Centrifuge for 10 min and filter the supernatant through a filter membrane for further use. Throat Swab: Place the throat swab in a 1.5 mL sterile centrifuge tube, add 500 μL of PBS, and vortex for 1 min. Discard the swab, then centrifuge at 3000 rpm/min for 5 min. Take the supernatant for further use. Tissue Sample: Take 1 g of thawed tissue, chop it into small pieces, and add 2 mL of PBS to grind and prepare tissue homogenate, at 8000 rpm/min. Centrifuge for 5 min and take the supernatant for further use. Fecal Swab: Place the fecal swab in a 1.5 mL sterile centrifuge tube, add 500 μL of PBS, and vortex for 1 min. Discard the swab, then centrifuge at 3000 rpm/min for 10 min. Take the supernatant for further use. Pig Milk: After thawing and refreezing the milk sample twice, centrifuge at 12,000 rpm/min for 5 min at 4 °C. Take the supernatant for further use. For blood samples, no pre-processing is required.

**Table A3:** Performance comparison results.

Method	Target Gene	Sensitivity/ Amplification Efficiency (E:90–110%)	Amplification Time	Add Sample Steps	Specificity
The method developed in this study	M gene	3.12 × 10^0^ copies/μL 100 TCID_50_/μL E: >90%	About 30 min	1	High
Qiu et al., 2020 [6]	NSP2/ORF5	10^1^ copies/μL E: <90%	About 98 min	5	High
Chen et al., 2019 [55]	M gene/ N gene	3.9 × 10^1^ copies/μL 5.6 (10^0.75^) TCID_50_/mL E: >90%	About 160 min	4	High
Ma et al., 2024 [58]	N gene	1.33 × 10^2^ copies/μL E: >110%	About 35 min	4	High
Amer et al., 2023 [57]	ORF4-6	10^1^ copies/μL E: >90%	About 49 min	3	High
Wang et al., 2024 [52]	ORF6	2.8 × 10^1^ copies/μL E: <90%	About 65 min	4	High
Fornyos et al., 2022 [59]	ORF5	10^1^ copies/μL E: =90%	About 70 min	5	High
