# Supplementary material for: A Simple and Sensitive RT-qPCR Technology for Rapid Detection of Porcine Reproductive and Respiratory Syndrome Virus
Source: Vet Sci. 2025 Jan 7;12(1):26. doi: 10.3390/vetsci12010026 (PMC11768678; doi:10.3390/vetsci12010026)
Supplement: Supplementary file 1 [file vetsci-12-00026-s001.zip › vetsci-3294931-supplementary.pdf]

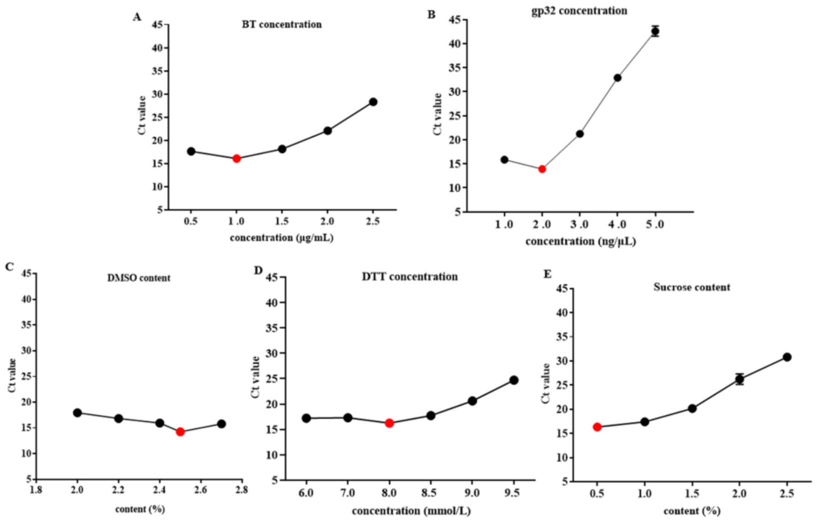

Figure S1. Optimization results of enhancer concentration in full pre-mix system**.** A: The final concentration of BT was 1 μg/mL, there was the best amplification result (Ct mean: 16.074). B: The final concentration of gp 32 was 2 ng/μL, there was the best amplification result (Ct mean: 13.909). C: The content of DMSO was 2.5%, there was the best amplification result (Ct mean: 14.244). D: The concentration of DTT was 6 mmol /L, there was the best amplification effect (Ct mean: 16.243). E: The Sucrose content was 0.5%, there was the best amplification effect (Ct mean: 16.345)

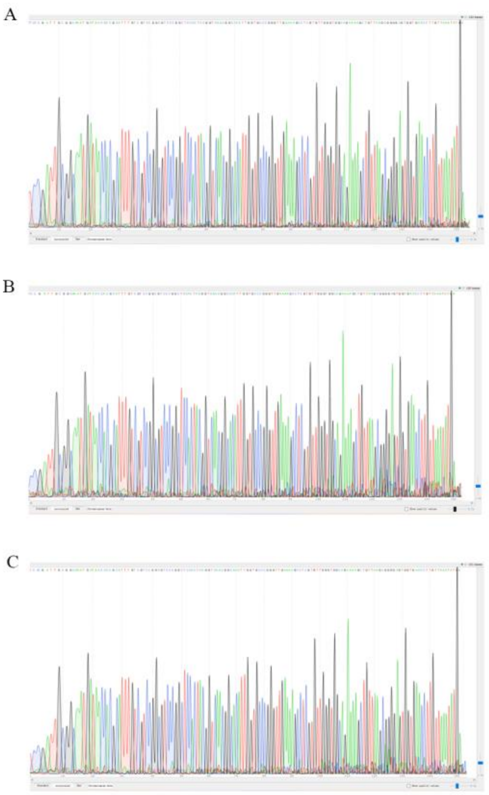

Figure S2. Results of the Sanger gene sequencing of the samples PCR products. A, B and C are gene sequencing results of PCR products from three serum samples, respectively.

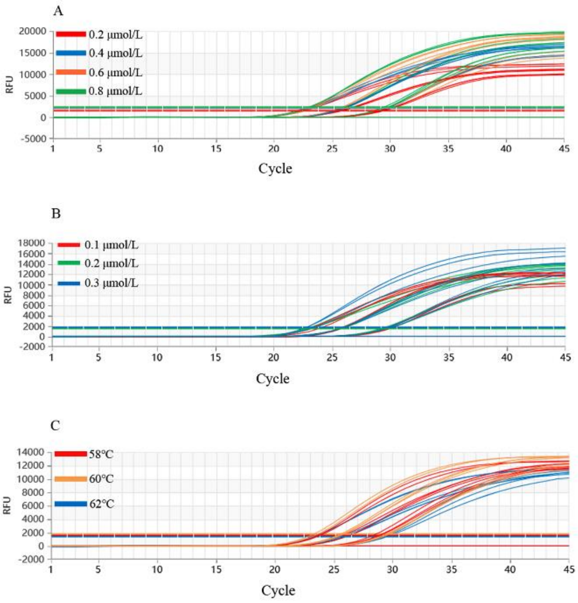

Figure S3. Optimization of the PRRSV RT-qPCR reaction system. A: Optimization results of primer concentration. The red, blue, yellow and green lines are the detection results of the primers at the concentration of 0.2 μmol/L, 0.4 μmol/L, 0.6 μmol/L and 0.8 μmol/L, respectively. B: Optimization results of probe concentration. The red, green, blue lines are the results of the detection results of the probe at the concentration of 0.1 μmol/L, 0.2 μmol/L, 0.3 μmol/L, respectively. C: Optimization results of denaturation temperature. The red, yellow, blue lines are the results of the detection results of the denaturation temperature of 58℃, 60℃, 62℃, respectively.

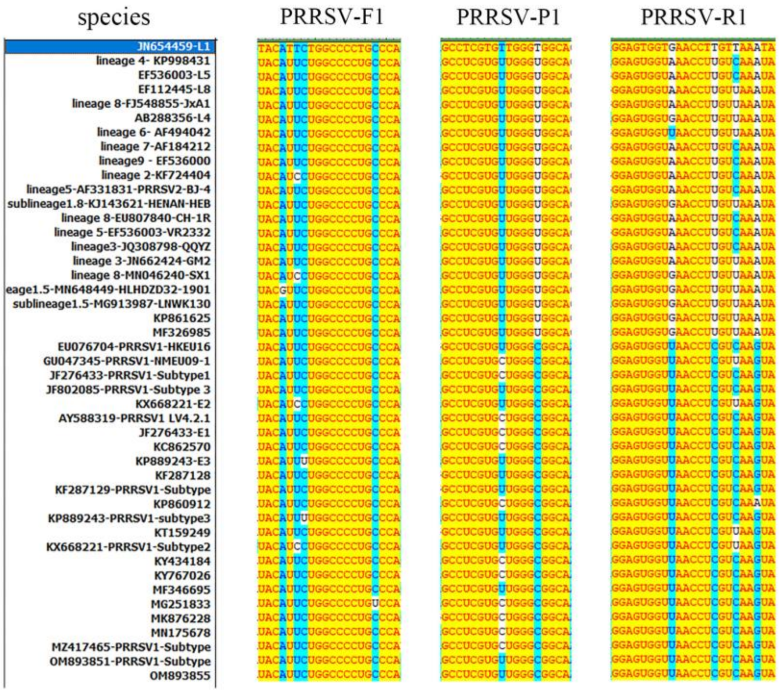

Figure S4. Sequence alignment results of PRRSV
